# Interactions Between Temperature Variability and Reproductive Physiology Across Traits in an Intertidal Crab

**DOI:** 10.3389/fphys.2022.796125

**Published:** 2022-03-08

**Authors:** Emily K. Lam, Metadel Abegaz, Alex R. Gunderson, Brian Tsukimura, Jonathon H. Stillman

**Affiliations:** ^1^Estuary and Ocean Science Center, San Francisco State University, Tiburon, CA, United States; ^2^Department of Integrative Biology, University of California, Berkeley, Berkeley, CA, United States; ^3^Department of Biology, San Francisco State University, San Francisco, CA, United States; ^4^Department of Ecology and Evolutionary Biology, Tulane University, New Orleans, LA, United States; ^5^Department of Biology, California State University, Fresno, CA, United States

**Keywords:** thermal sensitivity, climate change, neurophysiology, reproductive ecology, behavioral thermoregulation

## Abstract

Thermal extremes alter population processes, which can result in part from temperature-induced movement at different spatial and temporal scales. Thermal thresholds for animal movement likely change based on underlying thermal physiology and life-history stage, a topic that requires greater study. The intertidal porcelain crab *Petrolisthes cinctipes* currently experiences temperatures that can reach near-lethal levels in the high-intertidal zone at low tide. However, the thermal thresholds that trigger migration to cooler microhabitats, and the extent to which crabs move in response to temperature, remain unknown. Moreover, the influence of reproductive status on these thresholds is rarely investigated. We integrated demographic, molecular, behavioral, and physiological measurements to determine if behavioral thermal limits varied due to reproductive state. Demographic data showed a trend for gravid, egg bearing, crabs to appear more often under rocks in the cooler intertidal zone where crab density is highest. *In situ* expression of 31 genes related to stress, metabolism, and growth in the field differed significantly based on intertidal elevation, with mid-intertidal crabs expressing the gene for the reproductive yolk protein *vitellogenin* (*vg*) earlier in the season. Furthermore, VG protein levels were shown to increase with density for female hemolymph. Testing for temperatures that elicit movement revealed that gravid females engage in heat avoidance behavior at lower temperatures (i.e., have a lower voluntary thermal maximum, VT_max_) than non-gravid females. VT_max_ was positively correlated with the temperature of peak firing rate for distal afferent nerve fibers in the walking leg, a physiological relationship that could correspond to the mechanistic underpinning for temperature dependent movement. The vulnerability of marine organisms to global change is predicated by their ability to utilize and integrate physiological and behavioral strategies in response to temperature to maximize survival and reproduction. Interactions between fine-scale temperature variation and reproductive biology can have important consequences for the ecology of species, and is likely to influence how populations respond to ongoing climate change.

## Introduction

There is an increasing need to understand the effects of temperature on biota given ongoing global change. Elevated temperature affects organisms at all levels of biological organization, and perturbations at each level can act on each other in complex ways (Chaui-Berlinck et al., 2004; Huey et al., 2012). Furthermore, physiological and behavioral sensitivity to temperature varies across different stages of the life cycle (Angilletta, 2009). One major life-history stage that influences temperature-dependent behavior in females is gravidity or egg bearing (Cree and Hare, 2016). For example, when females of the oviparous lizard *Podarics muralis* become gravid, changes in thermoregulatory behavior result in reduced body temperature and reduced distance to refugia (Brana, 1993). Many species also have strong oviposition site thermal preferences. When given the choice within a laboratory thermal gradient, female alpine newts prefer a narrow range of temperatures for oviposition (Dvořák and Gvoždík, 2009), and thermal oviposition site selection is likely an adaptive behavior that promotes optimal temperatures for offspring survival and development in the butterfly *Pyrgus armoricanus* (Eilers et al., 2013). Some organisms, such as the crabs that are the focus of this study, carry their broods. Thus, the female can precisely determine the thermal conditions of her eggs by moving to different microhabitats. The female preference-offspring performance hypothesis suggests that this behavior evolutionarily favors temperatures that are optimal for embryonic development (Jaenike, 1978). At the same time, there is a high cost of reproduction in marine invertebrates (Fernandez et al., 2000) and females must also consider temperatures that maximize their own performance (Scheirs et al., 2000).

Organisms remain within a thermally optimum range by eliciting escape reflexes to avoid thermal stress (Lagerspetz and Vainio, 2006). The rate of neural firing is highly temperature dependent and could trigger heat avoidant movement. The voluntary thermal maximum (VT_max_) is an upper thermal tolerance limit where a temperature causes an organism to deliberately move to avoid a warming event (Cowles and Bogert, 1944; Camacho and Rusch, 2017). The thermosensory systems and physiological and molecular mechanisms that drive these behaviors are well studied, especially in model invertebrate organisms (Schafer, 2005; Barbagallo and Garrity, 2015). Kobayashi et al. (2016) found that even a single thermosensory neuron from *C. elegans* can memorize a temperature and influence downstream interneurons and thus may determine behavioral output. Many decapod crustaceans have nerve fibers that coordinate escape reflexes, and single neurons can command specific behavioral patterns in response to stimuli (Weirsma, 1946; Edwards et al., 1999). Nevertheless, relevant neurophysiological data linking thermosensory systems and behavior in crustaceans is sparse (Lagerspetz, 1973; Lagerspetz and Vainio, 2006). For example, lobster neurons change their rate of firing when exposed to different temperatures, but it is unclear if they trigger thermally driven behaviors (Konishi and Kravitz, 1978). Neural functions are the physiological basis for thermal escape behavior in crabs (Lagerspetz, 2000). Neural control of temperature selection may be mediated by thermoreceptors or thermosensitive neurons; however, little is known about the location or mechanisms used to sense temperature in crustaceans. Moreover, porcelain crabs have thermal plasticity in neural system performance thresholds after thermal acclimation and exhibit interspecific variation in nerve thermal tolerance (Miller and Stillman, 2012), but the behavioral repercussions and ecological outcomes of this plasticity are unknown.

The rocky intertidal zone is thermally heterogeneous and animals that inhabit the high-intertidal zone experience longer, more frequent exposure to extreme temperature compared to animals in lower intertidal zones (Evans, 1948; Helmuth, 2006). High-intertidal organisms also appear to have limited physiological plasticity to cope with warming (Stillman, 2003), such that rising temperatures may force these species to move down the shore to cooler and more stable intertidal zones (Stillman and Somero, 2000). However, they are more likely to encounter predation risks lower in the intertidal zone (Connell, 1961), as well as higher intraspecific densities and competition. Crowding stress can lead to lowered reproductive output and promote a negative relationship between gonad and hepatopancreas mass which is responsible for reproductive nutrient storage and associated with energetic limitations in reproductive individuals (Kennish, 1997; Thomson et al., 2010; Griffen et al., 2011; Zanette et al., 2011).

The reproductive protein vitellogenin (VG) is a useful marker for estimating reproductive state within populations, including how reproductive physiology is altered by changing environmental conditions. VG is found in all egg laying (oviparous) vertebrates and invertebrates, and functions as a precursor to the vitellin (VN) yolk protein which is necessary for ovarian maturation and embryonic development (Tsukimura, 2001; Liu C. et al., 2015). Vitellogenin production has been shown to increase during cooler months in intertidal crabs (Salas, 2017). Furthermore, levels of *vg* gene expression can change in response to acute stressors such as crowding or predator presence, and can correlate with egg yolk availability during embryonic development and act as a marker for reproductive output (Lethimonier et al., 2000; Schreck et al., 2001).

The anomuran porcelain crab *Petrolisthes cinctipes* inhabits the mid- to high-intertidal zone on rocky shorelines along the Pacific coast from British Columbia to Southern California. *P. cinctipes* currently lives at the upper limit of its physiological thermal range (Stillman and Somero, 2000) and has limited plasticity to physiologically buffer itself against warming (Stillman, 2003). *P. cinctipes* can also experience fluctuations in temperature of up to 20°C in a period of 6 h (Stillman and Somero, 1996; Gunderson et al., 2019). Therefore, it is likely that these crabs will need to behaviorally thermoregulate by moving to different microhabitats in order to avoid overheating under future warming (McGaw, 2003; Gunderson et al., 2019).

Using *P. cinctipes* as a model system, numerous investigations were conducted that span from the field to the lab to develop an integrative picture of how thermal variation affects populations with a focus on reproductive physiology. The common thread through all of the studies is the connection between physiological state and population demographics under different thermal conditions and species density scenarios. Demographic field surveys were used to determine how crab density and female gravidity vary along the intertidal thermal gradient. To test whether the occupation of different intertidal elevations influences the physiological state of crabs, *in situ* expression of genes related to stress, metabolism, development, and reproduction, including vitellogenin (*vg*) expression, were measured. Given that crab density differences were found with intertidal elevation, laboratory experiments were conducted to determine if *vg* expression levels or VG protein concentration were affected by density. To determine if reproductive state influences temperature-dependent behavior, heat avoidance behavior was measured in gravid and non-gravid females. To identify mechanisms underlying these behaviors, the physiological thresholds of neural thermal sensitivity were experimentally tested to detect correlations with heat avoidance behaviors. Thus, these studies present a holistic approach to coupling organismal physiology to ecological conditions within an environmental change context.

## Materials and Methods

### Demography and Habitat Temperature

Demographic data and temperature data were collected from June 19, 2015, through December 12, 2016, on a south-facing boulder shore just south of Fort Ross State Park on the northern California coast (38.5143°N, 123.2438°W; Gunderson et al., 2019). The California coast has mixed semidiurnal tides characterized by two high tides and two low tides of unequal amplitude each lunar day. Following Gunderson et al. (2019), the study area was categorized into two broad intertidal zones: mid-intertidal zone (MIZ; *n* = 12; mean intertidal elevation = 0.00 m; intertidal elevation range = −0.32 to 0.23 m), and high-intertidal zone (HIZ; *n* = 12; mean intertidal elevation = 0.80 m; elevation range = 0.31–1.10 m). Elevations for MIZ and HIZ are relative to mean lower low water (MLLW). Rock intertidal elevations were calculated using ground survey methods that employ yard sticks and a basic theodolite (Mason et al., 2000). Reference points for rock height measurements were set by assigning the observed lower low water level on a calm day as the lower low water level predicted for Fort Ross on that day based on tide charts. The length and width of each rock was measured, and rocks ranged in size from 568 to 2,671 cm^2^ surface area.

To sample *P. cinctipes* demographics, rocks were marked with unique identification numbers using Z-spar marine epoxy within the HIZ and MIZ transects. Temperature was monitored under rocks within the transects using temperature data loggers (iButton DS1921G, Dallas Semiconductor) that were placed inside waterproof brass casings (23 × 25 mm) and affixed to the center of the underside of rocks with Z-spar marine epoxy. For each sampling event marked rocks were flipped over, all crabs that had been underneath were collected, and were placed in a temporary holding container with fresh sea water. Eleven to fifteen rocks were sampled in the MIZ and 9–20 rocks in the HIZ each trip. Sampling occurred every 2 weeks when low tides permitted access to the site. The HIZ was sampled more frequently than the MIZ because the HIZ rocks were exposed more often and were therefore more frequently accessible for sampling. Demographic data are presented as density in terms of number of crabs per m^2^ of rock area sampled on a given date, as well as the proportion of rocks where gravid females were present.

### *In situ* Gene Expression Patterns of *Petrolisthes cinctipes* in the Field

From July 6th through December 22nd, 2015, crabs were collected from the HIZ and MIZ to measure expression of genes related to reproduction, physiological stress, and metabolism. This sampling period encompassed both low reproductive periods and peak reproductive periods as well as seasonal changes in temperature. Crabs were sampled from the HIZ on 11 occasions and the MIZ was sampled during six occasions because of the reduced accessibility of the MIZ. In sum, gene expression was measured in 160 individuals (HIZ *n* = 104; MIZ *n* = 56) split between females and males (female *n* = 82; male *n* = 78). Crabs were collected by flipping rocks adjacent and similar to, but not within, the HIZ and MIZ transects so that animals were not removed from the transects themselves. Upon collection, animals were immediately frozen on dry ice, were transported back to the laboratory on the same day and were stored at −80°C until RNA extraction.

Total RNA was extracted from the bodies of the crabs following the protocol outlined in Gunderson et al. (2017). Briefly, the walking legs and claws of the frozen crabs were removed, and the remaining bodies were ground to a powder in liquid nitrogen with a mortar and pestle. RNA was extracted from the powder using guanidine isothiocyanate extraction (Chomczynski and Sacchi, 1987) with Tri Reagent (Molecular Research Center, United States) according to the manufacturer’s protocol. Tri Reagent was added to each 50 mg powder sample along with nuclease-free stainless-steel beads and shaken in a TissueLyser (Qiagen) at 30 Hz for 10 min. Phase separation was performed with BCP (Molecular Research Center, United States), and isopropanol and a high salt buffer were used to precipitate the RNA. The RNA was washed twice with 75% ethanol and dissolved in 50 μL of RNase free water. RNA quality was assessed with A260/280 ratios and 1% agarose gel electrophoresis with ethidium bromide staining. RNA was stored at −80°C.

The measurement of target and housekeeping gene expression was conducted by the University of California, San Francisco Center for Advanced Technology using the Nanostring platform (Geiss et al., 2008). Nanostring generates read counts for each gene from each sample without cDNA synthesis or PCR amplification. 25 μL of RNA per sample at a concentration of 100 ng/μL was provided to generate expression data of 31 target genes and eight housekeeping genes (Table 1). Target genes were chosen based on their association with important biological processes including stress physiology (e.g., *heat-shock proteins, V-type proton ATPases*, genes associated with ubiquitination), metabolism (e.g., *cytochromes, acyl-coa synthetase*), growth (e.g., *cuticular proteins*), and reproduction (e.g., *vitellogenin, vitelline egg coat protein*). Nanostring probes were designed from cDNA (Tagmount et al., 2010) and RNA-seq (Armstrong and Stillman, 2016) transcriptomic datasets for *P. cinctipes*. Gene expression was standardized relative to the expression of internal positive controls (for target and housekeeping genes) and housekeeping genes (for target genes only) for each individual. A positive control correction factor was generated for each individual by dividing the geometric mean of positive controls for the individual by the geometric mean of positive controls across all individuals. The counts of all genes for that individual were then multiplied by this factor. Target genes for an individual were standardized to housekeeping genes in a similar manner, whereby target gene expression was multiplied by a correction factor taken as the geometric mean of housekeeping gene expression for that individual divided by the geometric mean of housekeeping gene expression across all individuals.

**TABLE 1:** Gene annotations, type (housekeeping (HK) or target), and gene function for sequences used in *in situ* gene expression assays.

Annotation	Type	Function
Actin	HK	–
Tubulin	HK	–
WD repeat-containing protein 5	HK	–
Partitioning defective 6 homolog gamma	HK	–
Paired amphipathic helix protein Sin3a	HK	–
U2 snRNP-associated SURP motif-containing protein	HK	–
Poly (U)-binding-splicing factor half pint	HK	–
Aldehyde Dehydrogenase, dimeric NADP-preferring	HK	–
Acyl-coA synthetase	Target	Lipid metabolism
WAP four-disulfide core domain protein 5	Target	Immunity
AMPK-5	Target	Lipid and carbohydrate metabolism
Phosphoenolpyruvate carboxykinase	Target	Carbohydrate metabolism
Hsp40	Target	Stress response
Complement C1q-like protein 4	Target	Immunity
SIR-2	Target	Cell cycle regulation
Retinitis pigmentosa GTPase regulator	Target	Development
Arginine kinase	Target	Amino acid metabolism
Hsp90 alpha	Target	Stress response
Epididymal secretory glutathione peroxidase	Target	Lipid metabolism/stress response
I-connectin	Target	Muscle function
AMPK-2	Target	Lipid and carbohydrate metabolism
Hsp83	Target	Stress response
Fatty acid-binding protein	Target	Lipid metabolism
Cuticle protein AM1159	Target	Development/molt
Cytochrome P450 2L1	Target	Oxidative metabolism
Cuticle protein Amp1a	Target	Development/molt
V-type proton ATPase subunit G	Target	Proton regulation
Endocuticle structural glycoprotein SgAbd-3	Target	Development/molt
Hsp70	Target	Stress response
Troponin I	Target	Muscle function
Cuticle protein AM/CP1114	Target	Development/molt
E3 ubiquitin-protein ligase UBR4	Target	Protein catabolism
V-type proton ATPase subunit D 1	Target	Proton regulation
V-type proton ATPase 116 kDa subunit	Target	Proton regulation
Cytochrome b-c1 complex subunit 8	Target	Oxidative metabolism
Ral guanine nucleotide dissociation stimulator	Target	Signal transduction
Ubiquitin-conjugating enzyme E2 J1	Target	Protein catabolism
Vitelline egg coat protein	Target	Reproduction
Vitellogenin (common and unique)	Target	Reproduction

### Experimental Animal Collection and Maintenance

*P. cinctipes* specimens were collected at Fort Ross State Park and transported in coolers with aerated seawater to the Estuary & Ocean Science Center in Tiburon, CA on the same day. For common garden conditions, specimens were held in a controlled 4,000 L flow-through recirculating seawater system at a density of 82 crabs/m^2^, an ambient temperature of 13 ± 0.5°C, and a salinity of 33 ± 3 ppt for 2–6 weeks unless specified elsewhere. Specimens were fed approximately a 1:10 dilution of Reed Mariculture Inc. Shellfish Diet every 2–3 days. Crabs were collected throughout the year, during both reproductive and non-reproductive seasons, from rocks that were not in our demographic transects. These crabs were collected for the following physiological experiments.

### Density Effects on Vitellogenin Expression and Protein Concentration

Non-gravid female crabs were collected during the end of the reproductive season (February and March 2017). The day after collection, crabs were individually housed in 20 × 7.6 cm acrylic cylinders with a mesh bottom and held in the recirculating seawater aquarium system for a 2-week common garden acclimation period. Cylinders had a continuous flow of water and were manually flushed daily to remove waste. Following acclimation, crabs were randomly placed in “high” (787 crabs/m^2^, *n* = 15) or “low” (250 crabs/m^2^, *n* = 13) density treatments. Treatment densities were based on a range of observed densities of *P. cinctipes* in the field. The low-density value was determined from the average of the lower quartile of observed density at the Fort Ross field site (Gunderson et al., 2019). The high-density value was determined based on field observations of *P. cinctipes* in Donahue (2004). Target densities were achieved by placing 14 individuals within 22 × 22 × 1.3 cm frame enclosures for the high-density treatment, and 10 individuals within 13.3 × 13.3 × 1.3 cm frame enclosures for the low-density treatment.

RNA was extracted from crab bodies and protein was extracted from hemolymph. Whole crabs and hemolymph samples were flash frozen in liquid nitrogen and stored at −80°C after a 2-week experimental period on March 16th and April 27th, 2017. To determine the expression level of vitellogenin, the walking legs and claws of the frozen crabs were removed, and crab bodies were ground to powder in liquid nitrogen using mortar and pestle. RNA was extracted using Trizol as described in Gunderson et al. (2017). A NanoDrop Spectrophotometer (A260) and 1% agarose gel electrophoresis stained with ethidium bromide were used to determine RNA quantity and quality. RNA was reverse transcribed to first strand cDNA using the iScript Reverse Transcription SuperMix kit (Bio-rad, kit cat. No. 170-8841) following the manufacturer’s instructions. *Actin* and α*-tubulin* were used as reference genes and used to normalize against *vitellogenin* (*vg*). Vitellogenin (VG) protein concentration for *Petrolisthes* hemolymph was determined using a competitive enzyme-linked immunosorbent assay (ELISA) protocol as described in Delmanowski et al. (2017). Hemolymph was drawn with a 27-gauge needle and 1 mL syringe and was transferred to a 1.5 mL Eppendorf tube at a 1:1 dilution with hemolymph buffer (0.1 M NaCl, 0.05 M Tris, 1 mM EDTA, and 0.1% Tween-20, pH 7.8). A subset of individuals used in *vg* analysis had hemolymph drawn prior for protein concentration analysis.

### Behavioral Heat Avoidance: Thermal Sensitivity to Gravid State

We compared heat avoidance behavior in gravid (GF; *n* = 62) and non-gravid female crabs (NGF; *n* = 30) exposed to a thermal ramp while partially immersed in seawater. These crabs did not undergo density experiments and were held in common garden conditions for 2 weeks prior to experimentation. Experimental crabs had a carapace width between 8 and 15 mm. Heat avoidance was measured as the voluntary thermal maximum (VT_max_), recorded as the temperature at which a crab exited a temperature-controlled chamber during a thermal ramp (Cowles and Bogert, 1944; Camacho and Rusch, 2017).

The temperature chamber was constructed using a petri dish (100 × 15 mm) filled with aerated, filtered seawater nested in an aluminum block (15 × 15 cm) fitted with internal copper tubing (outer 3/8”) and connected to a water bath by flexible PVC (inner 3/8”; Supplementary Video 1; Gunderson et al., 2019). The aluminum block and tubing were insulated with foam and walking surfaces were covered in an adhesive grip tape to prevent crabs from slipping (Jessup the Original ^®^). The chamber was covered with a ceramic dish elevated 4 cm above the surface to provide shelter and simulate the dark under-rock environment. The water temperature was monitored with a digital thermometer (Omega model HH603A, type T, sensitivity 0.1°C). Initially, the experiment was conducted using a temperature chamber for an individual crab and later a chamber which assayed 6 crabs simultaneously was used to increase throughput. The high throughput temperature chamber was built using an insulated aluminum block (36 × 23 cm) with six small, nested petri dishes (60 × 15 mm). Each dish was filled with aerated seawater and was covered with an opaque plastic lid for shelter and to prevent disturbance from neighboring crabs. Water temperature was recorded using a multichannel thermocouple (Omega model HH378, type K).

In both devices, crabs were placed in the dish at 13°C and were contained with a blockade for 10 min at a constant temperature. The blockade was removed, and the temperature was ramped by a controlled water bath at 0.5°C per minute. The VT_max_ was recorded as the temperature of the water when the crab exited the dish, and all appendages were in contact with the grip tape. Control crabs (n = 80) were similarly held at 13°C for 10 min before the blockade was removed, then the temperature remained at 13°C for 30 min, which was the maximum time of any treatment trial.

### Thermosensory Behavior and Neural Thermal Performance

To demonstrate that crabs sensed temperature with their legs, behavioral responses to isolated thermal stimulation on the walking legs of (*n* = 7) crabs were studied. Seawater was dropped on the left 3rd walking leg with a 27 G × 1/2 syringe in approximately 1°C intervals at temperatures between 17 and 39°C (Supplementary Video 2). Water temperature was measured with a digital thermometer (Omega model HH603A, type T, sensitivity 0.1°C) immediately before administering a drop to the crab leg. Experiments were conducted with crabs placed in a petri dish in air at room temperature (21–24°C). When a drop of water was placed on the crab leg, the behavioral response of the crab was recorded (i.e., moved away or did not move away).

Action potential propagation in afferent nerve fibers exposed to a thermal ramp was measured to quantify thermal sensitivity of thermosensitive neural systems in crab walking legs. Neural thermal performance was recorded in a group of gravid (GF, *n* = 9) and non-gravid female crabs (NGF, *n* = 10) crabs 7–16 days after being assayed for VT_max_ to determine if there is a link between heat avoidance behavior and neural heat sensitivity and an effect of reproductive state. These individuals did not experience thermal stimulus isolated to their walking leg as in the previous experiment. Spontaneous field potential propagation was determined in nerve fibers of the 3rd walking leg during a thermal ramp following the protocol from Miller and Stillman (2012). Briefly, the left 3rd walking leg was removed by gently tapping the joint between the coxa and basischium with forceps and allowing it to autotomize. Sensory neurons were isolated by cutting away the basischium article and separating the merus from the carpus. Using this method, the nerve bundle is exposed and remains attached to the distal portion of the walking leg. The dissected nerve preparation was placed in a temperature-controlled petri dish filled with seawater at 13°C.

Measurements were taken on an anti-vibrational table, in a grounded faraday cage with the lights turned off to reduce electrical noise, using a hand-pulled glass Ag/AgCl suction electrode. A glass capillary tube was pulled with an oxygen acetylene torch, cut at the appropriate point to produce the right diameter opening, and the cut tip was flame polished. Silver wires were submerged in bleach (6% sodium hypochlorite) for 30 min to chloride the wire. An Ag/AgCl wire was placed in the glass capillary tube and a reference Ag/AgCl wire was wrapped around the tube. The electrode was attached to a Grass P55 A.C. pre-amplifier at 1,000× amplification with the low-pass filter at 30 Hz and the high-pass filter at 1 kHz to distinguish neural spikes. The glass Ag/AgCl electrode was filled with seawater and negative pressure was applied with a 10 mL syringe to suction a loop of the nerve into the tip of the electrode. The output was recorded using a Power Lab 15T (ADInstruments) and continuous measurements were recorded using LabChart software (ADInstruments Chart v.8.1.5).

**FIGURE 1:**
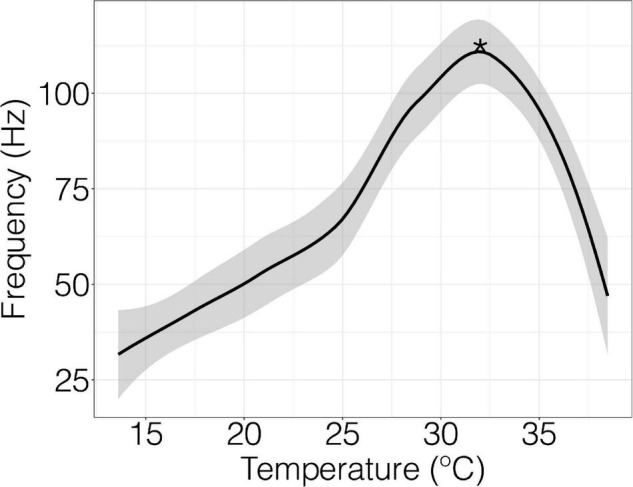
Field potential frequency and temperature in an individual crab with raw data fitted by Loess function. Peak firing temperature is denoted by the asterisks (*). The detection threshold of the recording was 200 mV at a rate of 40 k/s. The temperature was ramped at 0.5°C per minute.

Spontaneous field potentials of nerve fibers were recorded in mV with the detection threshold set to 200 mV at a rate of 40 k/s (Supplementary Figure 2). The temperature was ramped at 0.5°C per minute controlled by a Lauda proline RP 855 water bath. The frequency of field potential firing as a function of temperature provided a thermal performance curve (Figure 1). Recordings began immediately after the nerve was fixed to the suction electrode and continued until nerve function ceased. Thermal performance curves were obtained by using LabChart software extension spike histogram and the built-in macros to extract field potential frequency (Hz) and temperature data. The initial point at which firing frequency showed a significant change from baseline was considered the initial firing temperature and the point at which firing frequency was the highest was called peak firing temperature. Initial firing temperature was extracted by identifying frequency values that are significantly different from baseline using a sliding window function. Optimal peak firing temperature was determined from thermal performance curve using R software (v.4.1.1; R Core Team, 2017) and applying the “loess” function. Neural profiles were discarded if a peak temperature was not generated because of diminished signal if the nerve slipped from the suction from the electrode.

### Statistical Analyses

Statistical analyses were conducted in R software (v.4.1.1; R Core Team, 2017). Differences in average responses were examined with Welch’s two sample *t*-test in laboratory physiology and behavioral experiments. Expression data were condensed using principal components analysis on log-transformed standardized transcript counts. Principal component data were used in linear models to test for associations between gene expression and day of the year (i.e., time), sex, intertidal zone, and their interactions. Following Crawley (2010), we started with full models with all interactions and simplified models by removing non-significant interaction terms. All gene expression standardization and analyses were conducted in R (R Core Team, 2017). A linear mixed effects model (LME) was used to compare VT_max_ in GF and NGF where date and trial were included as random effects. A generalized linear mixed effects model (GLMM) was used to determine the response to discrete thermal stimulus on the crab walking leg and repeated measures were included as a random effect. Linear regression was used to define the relationship between peak sensory nerve firing and VT_max_ in female crabs.

## Results

### Demography and Habitat Temperature

**FIGURE 2:**
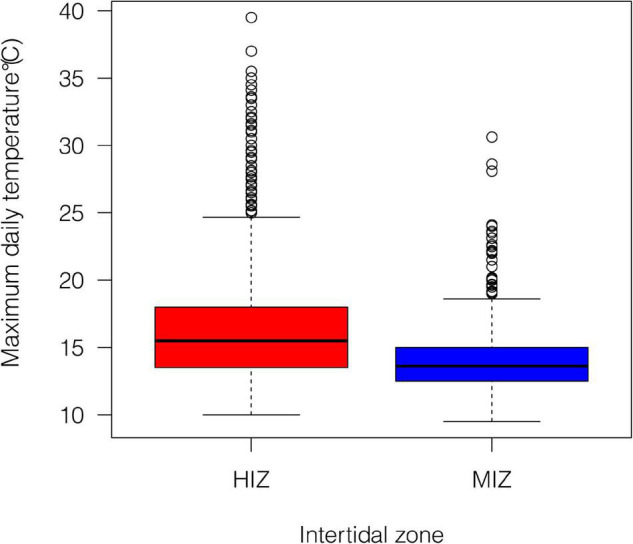
Summary of daily maximum temperature under rocks within our study site at different intertidal zones from July 2015 to December 2016. Data from Gunderson et al. (2019), where greater detail about the thermal conditions can be found.

**FIGURE 3:**
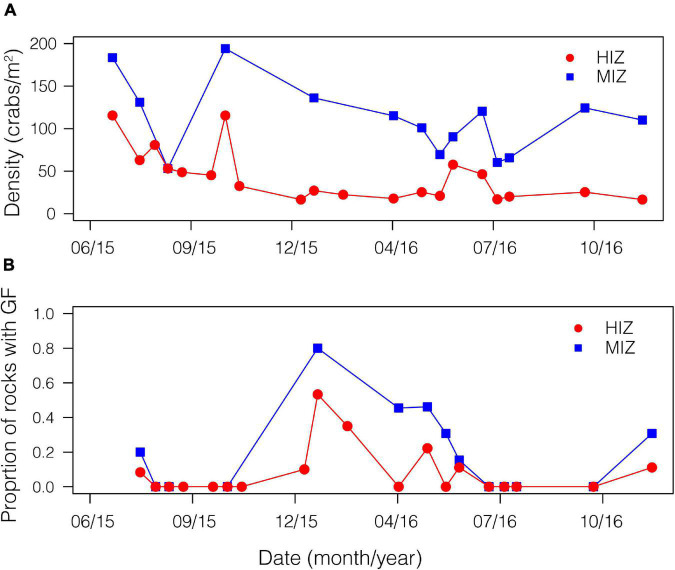
Demographic patterns by intertidal zone for *P. cinctipes.* On a boulder shore in northern California by time of year. **(A)** Density of crabs in the MIZ (mean ± SE: 108.6 ± 10.9 crabs/m^2^) and HIZ (mean ± SE: 43.4 ± 6.8 crabs/m^2^). **(B)** Proportion of rocks with gravid females.

Mean daily maximum temperature in the HIZ (16.4 ± 4.1°C) was 2.5°C higher than in the MIZ (13.9 ± 1.8°C) and the HIZ reached temperatures that were higher than 25°C more often than the MIZ (Figure 2; Gunderson et al., 2019). Crabs were consistently found at higher densities within the MIZ (mean ± SE: 108.6 ± 10.9 crabs/m^2^) than the HIZ (mean ± SE: 43.4 ± 6.8 crabs/m^2^; Figure 3A). The highest observed density under an individual rock was 697 crabs/m^2^ (Figure 3A). There was clear seasonality to *P. cinctipes* reproductive patterns. Gravid females were found from early winter through early summer, with the most gravid females found in winter (Figure 3B). There were more rocks in the MIZ with gravid females under them than in the HIZ (48 vs. 24%, respectively; Chi square test, *X*^2^ = 3.73, *p* = 0.054), but the proportion of females that were gravid did not differ between the MIZ and HIZ (0.49 vs. 0.43, respectively; Chi square test, *X*^2^ = 0.22, *p* = 0.637).

### *In situ* Gene Expression Patterns of *Petrolisthes cinctipes* in the Field

**FIGURE 4:**
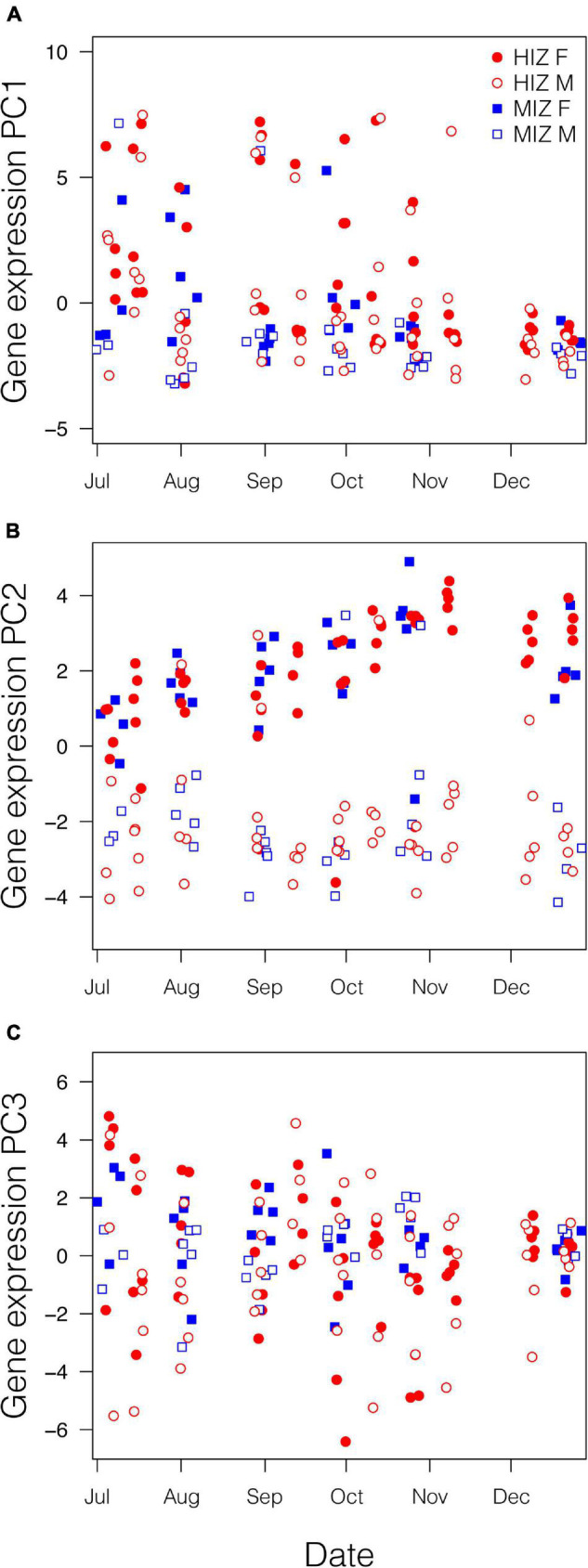
Patterns of gene expression for crabs in the field related to date, intertidal zone height, and sex. Gene expression data have been transformed using principal component analysis (see Supplementary Table 2). **(A)** The model explains 22% of the variation in PC1, **(B)** 66% of the variation in PC2 and **(C)** 4% of the variation in PC3. Samples from a particular sampling date are jittered so that all of the data points are visible.

Results from PC axes 1–3, each of which explain at least 10% of the variation in gene expression individually and collectively explain 60% of the variation, are presented here. The first expression principal component axis (PC1) explained 26% of the variability in gene expression. PC1 was most strongly associated with high expression of *hsp40*, *V-type proton ATPase subunits*, and *cuticular protein am1159* and *amp1a*, and low expression of *vitelline egg coat protein*, *arginine kinase*, and the molecular chaperones *hsp90* and *hsp70* (Supplementary Table 2). No interactions were found between sex, date, or intertidal zone (all *p* > 0.05), but all individual terms were significant (Table 2A, model explains 22% of the variation in PC1). Gene-expression PC1 decreased significantly over time from July to December in all crabs and was higher on average in the HIZ and in females (Table 2A and Figure 4A).

**TABLE 2:** Results of linear models for factors explaining variation in gene expression principal components for field collected crabs.

	df	SS	MS	*F*	*P*
**A. PC1**					
Date	1	141.8	141.8	21.2	<0.001
Intertidal zone	1	77.3	77.3	11.6	<0.001
Sex	1	36.8	36.8	5.5	<0.001
Residuals	156	1043.2	6.7		
**B. PC2**					
Date	1	20.2	20.2	8.7	0.004
Intertidal zone	1	0.0	0.0	0.0	0.915
Sex	1	639.2	639.2	275.5	<0.001
Date × Sex	1	23.8	23.8	10.3	0.002
Residuals	155	359.7	2.3		
**C. PC3**					
Date	1	4.7	4.7	1.1	0.289
Intertidal zone	1	17.4	17.4	4.2	0.042
Sex	1	6.5	6.5	1.6	0.213
Residuals	156	647.6	4.2		

The second expression principal component axis (PC2) explained 21% of the variability in gene expression. PC2 was most strongly associated with high expression of *vitellogenin*, *complement c1q-like protein 4*; and *wap domain protein 5*; and low expression of *retinitis pigmentosa gtpase regulator* and *hsp83* (Supplementary Table 2). There was a significant sex × date interaction associated with expression PC2, where PC2 increased in females on average from July to December in both the HIZ and MIZ but remained relatively constant for males; PC2 also had higher expression in females compared to males (Table 2B and Figure 4B; model explains 66% of the variation in PC2).

The third expression principal component axis (PC3) explained 13% of the variation in the expression data and was associated most strongly with high expression of *V-type proton ATPase subunits*, *troponin I*, and *cytochrome b-c1 complex subunit 8*; and low expression of *ampk-2*, *cuticle protein am/cp1114*, and *epididymal secretory glutathione peroxidase* (Supplementary Table 2). There were no interactions between factors with respect to PC3, though there was an effect of intertidal zone, with crabs in the MIZ having higher values than crabs in the HIZ (Figure 4C and Table 2C; model explains 4% of the variation in PC3).

**FIGURE 5:**
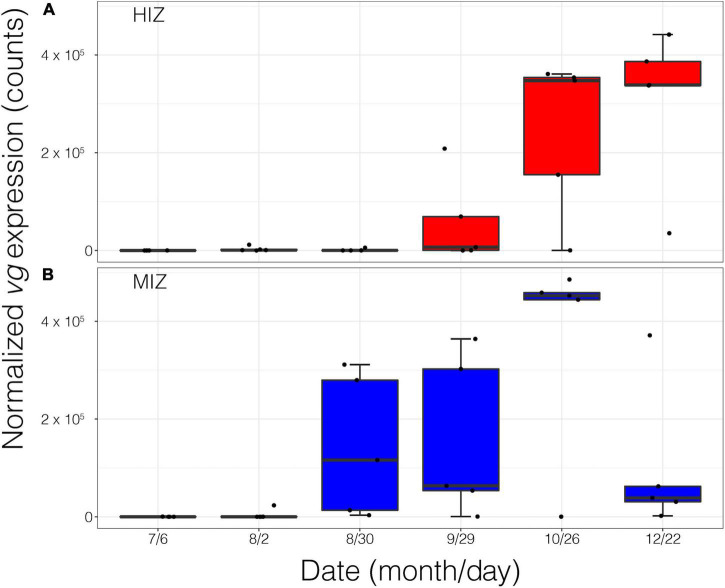
Normalized *Vitellogenin* gene expression of female crabs (*n* = 58) in the field from July 2016 to December 2016. **(A)** high-intertidal zone (HIZ) and **(B)** mid-intertidal zone (MIZ).

In the field, *vg* expression was seasonally elevated in female crabs (Figure 5). Intertidal height shifted vg temporally such that vg onset in female crabs from the HIZ did not occur until October and remained high through December (Figure 5A). MIZ female crabs began expressing vg in late August, peak expression was in October and expression began to decline in December (Figure 5B).

### Experimental Effects of Density on Vitellogenin Expression and Protein Concentration

**FIGURE 6:**
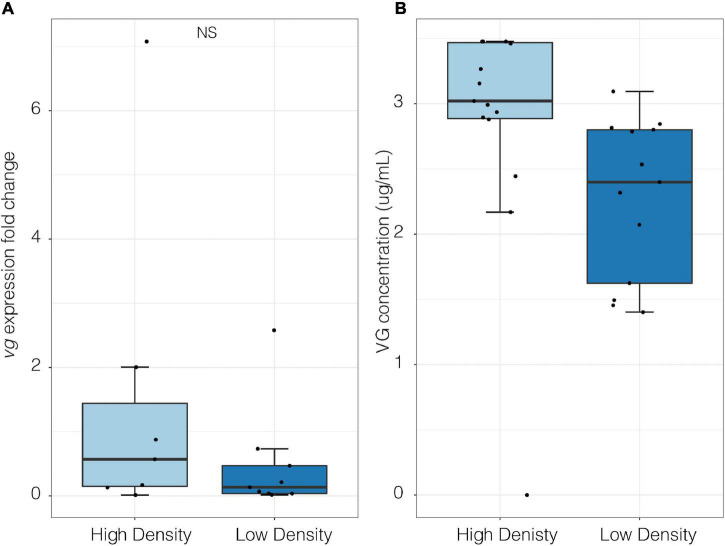
Molecular quantification of vitellogenin in female *P. cinctipes* from laboratory density experiments. **(A)** *Vitellogenin* gene expression under high (787 crabs/m^2^, *n* = 15) and low (250 crabs/m^2^, *n* = 13) densities (*p* > 0.05, high density mean = 0.62 ± 0.3 SE, low density mean = 0.212 ± 0.09 SE; Welch Two Sample *t*-test). **(B)** Vitellogenin protein concentration in hemolymph under high (787 crabs/m^2^, *n* = 15) and low (250 crabs/m^2^, *n* = 13) densities [Two-way ANOVA, *F*_(1, 26)_ = 0.05, *p* = 0.05]. Data are log transformed.

In the lab there was no significant difference in *vg* expression fold change between high- and low-density groups (*p* > 0.05, high density mean = 0.62 ± 0.3 SE, low density mean = 0.212 ± 0.09 SE; Welch Two Sample *t*-test; Figure 6A). However, under high density assemblages VG protein concentration was significantly higher than in low density assemblages (Two-way ANOVA, *F*_1,26_ = 0.05, *p* = 0.05; Figure 6B).

### Behavioral Heat Avoidance and Thermal Sensitivity to Gravid State

**FIGURE 7:**
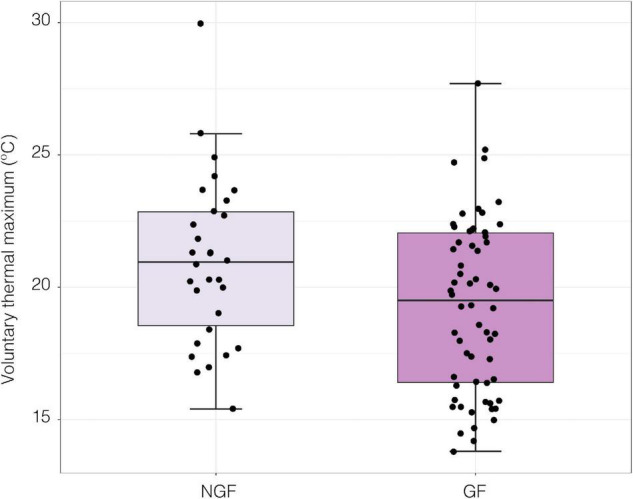
Voluntary thermal maximum (VT_max_) of gravid (GF; *n* = 62) and non-gravid female (NGF; *n* = 30) crabs under a thermal ramp of 0.5°C per minute [LME; *t*_(1, 17)_ = −2.24, *p* = 0.039].

When exposed to a thermal ramp, gravid females (GF) had significantly lower VT_max_ than non-gravid females (NGF; LME; *t*_1,17_ = −2.24, *p* = 0.039, Figure 7).

### Thermosensory Behavior and Neural Thermal Performance

**FIGURE 8:**
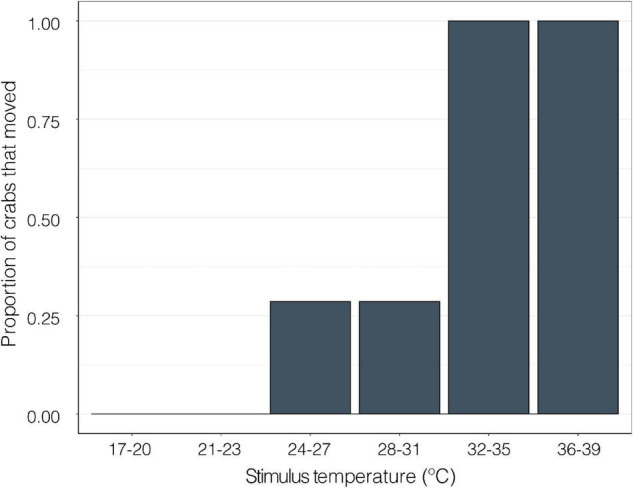
The proportion of crabs (GF; *n* = 7) that responded to isolated thermal stimulus from a drop of water on the distal portion of a walking leg in approximately 1°C intervals at temperatures between 17 and 39°C (GLMM; z = −11.30, *p* < 0.0001).

When a thermal stimulus (water drops of discreet temperatures) was applied to the walking legs of crabs, 0% of crabs moved at temperatures between 17 and 23°C. 29% of crabs moved at 24–31°C. 100% of crabs exhibited a behavioral response to the hot water at temperatures above 31°C (GLMM; z = −11.30, *p* < 0.0001, Figure 8).

When nerve fibers of the terminal segments of the walking leg were exposed to a thermal ramp, field potential rates increased slowly, reached a peak firing frequency, and then fell at temperatures beyond permissive thermal thresholds (Figure 1).

**FIGURE 9:**
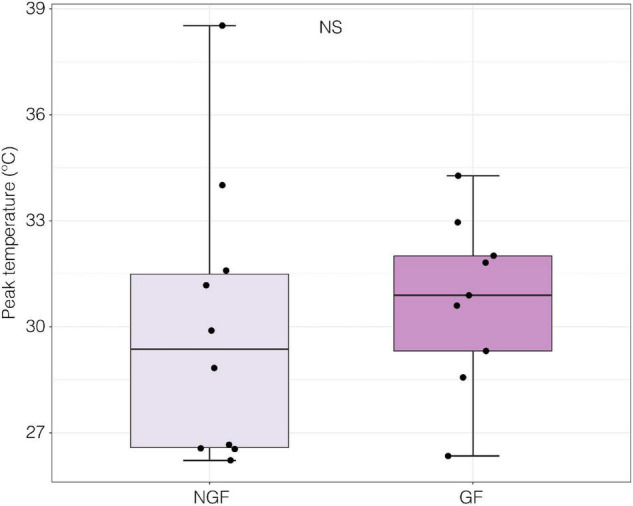
Peak firing temperature from field potential measurements of a nerve bundle from the distal walking leg in and gravid females (GF, *n* = 9) and non-gravid females (NGF, *n* = 10) exposed to a thermal ramp (*p* > 0.05, GF mean = 30.7 ± 0.80 SE, *F* mean = 30.0 ± 3.98 SE; Welch Two Sample *t*-test).

**FIGURE 10:**
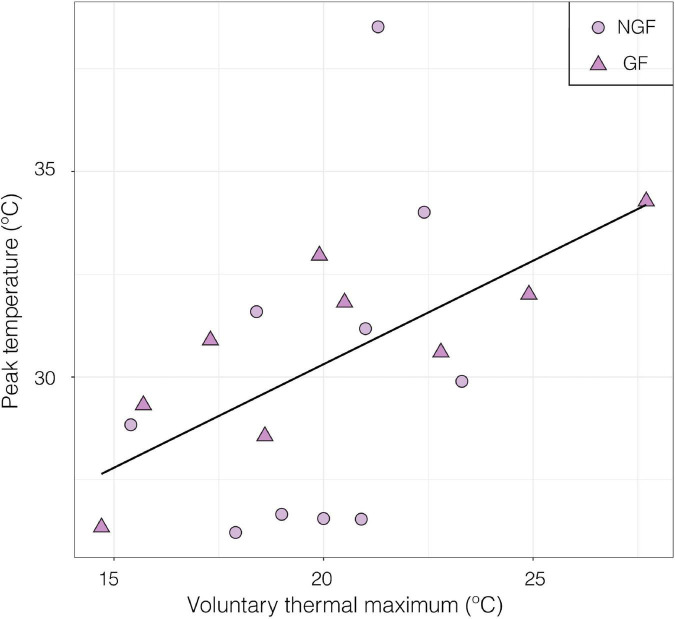
Relationship between the temperature of peak neural firing and Voluntary thermal maximum (VT_max_) of female porcelain crabs (gravid and non-gravid) exposed to a thermal ramp. Gravid females (GF, *n* = 9) are represented by triangles and non-gravid females (NGF, *n* = 10) are represented by circles (Slope y = 4.5 + 0.51x) [linear regression; *R*^2^ = 0.259, *F*_(1, 17)_ = 5.94 *p* ≤ 0.05].

Peak nerve firing temperature was not significantly different in gravid and non-gravid females (*p* > 0.05, GF mean = 30.7 ± 0.80 SE, *F* mean = 30.0 ± 3.98 SE; Welch Two Sample *t*-test) (Figure 9). In this subset of individuals where neural performance data was generated, there was also no significant difference in VT_max_ between gravid and non-gravid females (*p* > 0.05, GF mean 28.33°C ± 1.43 SE, *F* mean = 24.4°C ± 0.74 SE; Welch Two Sample *t*-test; Data not shown). However, the temperature of peak sensory nerve firing was positively correlated to VT_max_ in both gravid and non-gravid female crabs (slope y = 4.5 + 0.51x) [linear regression; *R*^2^ = 0.259, *F*_(1, 17)_ = 5.94 *p* ≤ 0.05] (Figure 10).

## Discussion

Thermal conditions in the intertidal zone fluctuate greatly in both time and space, which is likely to have major implications for the reproductive physiology of taxa living within intertidal habitats. This study took an integrative approach to understand how temperature variability affects the reproductive physiology of the intertidal crab *Petrolisthes cinctipes*, a species which can be exposed to physiologically damaging temperatures in the field (Stillman, 2003; Gunderson et al., 2019). The goal was to perform a set of interrelated studies that examined aspects of organismal physiology at cellular- and tissue-levels that were integrated with organismal behavior and ecological distributions across a range of environmental and ecological conditions.

### Demography of *Petrolisthes cinctipes* Across the Intertidal Zones

Intertidal zone organisms often show population structure along intertidal elevation gradients (Vermeij, 1972) and *P. cinctipes* is no exception. Crabs were less dense in the HIZ than in the MIZ (Figure 3A), a result that is perhaps not surprising given that the HIZ habitat that we sampled represents a distributional edge (i.e., near the maximum intertidal elevation of *P. cinctipes* at this site), while the MIZ habitat is an interior region of the distribution. Our results are consistent with observations within the intertidal and more broadly that densities are low at local distributional limits (Robles, 1987; Takada, 1996; Pardo and Johnson, 2005).

*P. cinctipes* at our site demonstrated clear seasonality in reproductive biology. Gravid females were found from winter through early summer, with the most gravid females observed during winter months (Figure 3B). Brood extrusion by females was preceded by upregulation of *vg*, the precursor molecule to the vitellin protein deposited in yolk (Tsukimura, 2001) in late summer and fall. This can be seen in gene expression PC2, which loaded highly for *vitellogenin* and increased over the season in females but not in males (Supplementary Table 2 and Figure 4B). These reproductive patterns are consistent with reproductive patterns observed for this species at a site approximately 160 km south on the California coast (Delmanowski et al., 2017). Intertidal zone height did not appear to influence whether a female became gravid, but female crabs in the MIZ had high levels of *vitellogenin* 2 months earlier than females in the HIZ. A greater proportion of rocks in the MIZ had gravid females than rocks in the HIZ. However, this may simply be due to differences in the density of crabs in each zone, as the proportion of females that were gravid in each zone did not differ.

### Expression of Stress and Reproductive Genes *in situ*

Spatial and temporal variation in abiotic conditions generated spatial and temporal variation in gene-regulation as organisms respond to prevailing conditions, as expected (Tagmount et al., 2010; Banni et al., 2011; Chapman et al., 2011; Lockwood et al., 2015). Consistent with previous studies that have shown differences in gene expression among individuals at different intertidal elevations (Gracey et al., 2008; Place et al., 2008; Clark et al., 2018) gene expression PC1 was associated with high expression of *hsp40*, *cuticle protein gene*s, and *V-type proton pump genes*, and low expression of *hsp70, hsp90, arginine kinase*, and *vitelline egg coat protein* (Supplementary Table 2). The lack of high expression of most heat-shock protein genes in the HIZ (Supplementary Figure 1) is somewhat surprising given that the HIZ reaches much higher temperatures than the MIZ (Gunderson et al., 2019), and high heat shock protein gene expression in the high- compared to the low-intertidal has been shown in other systems (Place et al., 2012). However, during sampling for gene expression in 2015, the mean maximum temperatures in the HIZ were rarely above the 25°C temperature threshold that has been measured in the lab for heat shock protein gene induction (Gunderson et al., 2019). Repeated sampling to target a period where mean maximum temperatures often exceed the 25^°^C threshold may result in higher heat shock protein gene induction, as seen in other studies.

Gene expression PC1 decreased over time in both intertidal zones, indicating that temperature may still play a role in driving these gene expression patterns without inducing stress, as mean and maximum temperatures in both zones decreased over the period for which expression data was collected (Figure 4A and Table 2). There was also a great deal of variation among crabs in PC1 in both zones (Figure 4A), which might be expected given the high degree of microclimatic variability among boulders (Gunderson et al., 2019) as fine-scale microclimatic variation can induce physiological differences in intertidal organisms over short distances (Jimenez et al., 2015).

Gene expression patterns often differ between sexes (Chang et al., 2011; Manousaki et al., 2014), and this was most apparent in *P. cinctipes* in gene expression PC2, with females having higher values than males in both zones. PC2 was most strongly associated with high expression of *vitellogenin*, *complement c1q-like protein 4*, and *wap domain protein 5 genes*, and low expression of *retinitis pigmentosa GTPase regulator* and *hsp83*. The high expression of *vitellogenin* in the fall and early winter precedes the egg extrusion that was observed in females in January through May (Figure 5) and is also commensurate with seasonal patterns of hemolymph vitellogenin concentrations observed in *P. cinctipes* on the California coast (Delmanowski et al., 2017). *Complement c1q-like protein 4* and *wap domain protein 5* are related to immune function (Amparyup et al., 2008; Du et al., 2010; Gerdol et al., 2015; Visetnan et al., 2017), and their greater expression in females is consistent with sex-specific differences in the expression of immune function genes seen in other crabs (Liu Y. et al., 2015). *Retinitis pigmentosa gtpase regulator (rpgr)* is a gene associated with retinal function in mammals (Rao et al., 2016). Nonetheless *rpgr* has been shown to have sex-specific expression in crustaceans, although usually with females having higher expression than males (Zeng et al., 2011; Peng et al., 2015); why females would have lower expression than males in *P. cinctipes* is unclear. HSP83 proteins have diverse functions and have sex-related expression differences in arthropods with males having higher expression than females (McAfee et al., 2017), though the consequences of sex specific expression are unknown. Overall, the sex-specific patterns of gene expression implicate genes that are important for female reproduction and can potentially be used as indicators of the reproductive state of individuals in the field.

Gene expression PC3 was associated with high expression of *v-type proton atpase subunits*, *troponin I*, and *cytochrome b-c1 complex subunit 8*, and low expression of *ampk-2*, *cuticle protein am/cp1114*, and *epididymal secretory glutathione peroxidase* (Supplementary Table 2). PC3 differed between intertidal zones, with higher values in the MIZ (Figure 4C and Table 2). However, the model explained only 4% of the variation in PC3, and therefore is of little value in identifying patterns of gene expression that differ between the zones.

### Vitellogenin Protein and mRNA Expression Levels in Female Crabs in High- and Low-Density Assemblages

The results from the density experiments did not support the hypothesis that crowding stress suppresses reproduction in porcelain crabs. High density assemblages were found to increase VG levels in the hemolymph. Elevated VG levels were similarly observed after a heat stress event in porcelain crabs (Salas, 2017). One possible explanation is that crabs are undergoing resorption of the oocytes, which is an established adaptive strategy to preserve and recycle nutrients under unfavorable conditions (Tuck et al., 1997; Becker et al., 2020). The ELISA system may not be able to detect the difference between VG and vitellin, the latter may have been released from resorbed oocytes, as the animal experiences stress. Another alternative is that the density used here may not have been high enough to elicit a strong enough stress response to suppress VG. With future warming, organisms may behaviorally thermoregulate resulting in local distribution shifts to cooler environments (Sunday et al., 2012). Range contractions often cause an increase in density and therefore we expect *P. cinctipes* habitat densities to increase as temperatures become more extreme. A follow-up study incorporating a larger range of densities and time series could help to understand the reproductive consequences of crowding. Previous density gradient studies have linked increased density to reduced numbers of eggs and offspring survival in snails. This negative relationship between density and fecundity has been observed across many taxa (Mangal et al., 2010).

Many factors influence the abundance of circulating VG in the hemolymph. Vitellogenin levels are related to oocyte developmental stage and have been shown to peak after spawning in the blue crab, *Callinectes sapidus* (Lee et al., 1996). Field experiments have reported an average hemolymph VG concentration of 23.1 μg/mL ± 6.4 SEM in April (Salas, 2017), which is the same month that hemolymph samples were collected in this study. Low levels of VG were found in the density experiments relative to the average levels observed in the field, which may suggest that hemolymph was sampled near the end of the reproductive season and crabs were not vitellogenic. The sampling may also have been associated with full moons, when *P. cinctipes* are not producing VG, whereas high levels of VG are synthesized during new moons (Salas, 2017).

### Behavioral Sensitivity to Temperature in Gravid Females

The behavioral heat avoidance results predict that gravid females should be found in cooler, more stable regions of their distribution. Although a greater proportion of rocks in the MIZ had gravid females (Figure 3B), intertidal zone height did not appear to have an effect on whether or not a female became gravid. Gravid females may not be avoiding the HIZ but rather may delay reproduction in the HIZ until temperatures are cooler, however, gravid crabs may not be able to maintain this strategy with future warming. Female crabs in the HIZ had high levels of vitellogenin later in the season compared to females in the MIZ (Figure 5), and thus presence in the HIZ could reduce fecundity. Gravid or reproductive females tend to seek refugia from predation to a higher degree than non-gravid crabs and *P. cinctipes* is more susceptible to predation when lower in the intertidal zone (Jensen and Armstrong, 1991); therefore it appears that the observed patterns are likely also driven by biotic factors.

There are several potential explanations for why gravid females may be more averse to high temperatures. Gravid females invest a substantial amount of energy on brood care in addition to reproductive output which place constraints on their available energy budget for activity (Brante et al., 2003). Therefore, they may have fewer energy reserves available to tolerate thermal stress. Reproduction itself is highly thermally constrained. For example, egg attachment and retention are temperature-controlled processes and in crustaceans, attachment failure has been reported at high temperatures (Waddy and Aiken, 1995; Fischer and Thatje, 2008). Additionally, temperature regulates ovarian maturation and egg laying where egg laying, or extrusion of the brood, only occurs at low temperatures (Aiken, 1969). Previous research on *P. cinctipes* shows that exposing late-stage embryos to a heat-shock of 30°C for 1 h causes a 20.3% reduction in brood survival (Yockachonis et al., 2020).

### Thermosensory Behavior and Neural Thermal Performance

Behavioral studies reveal that crustaceans can detect fluctuations in temperatures with great precision. For example, in lobsters, changes of 0.15°C can trigger a response (Crossin et al., 1998; Jury and Watson, 2000). This study confirmed that *P. cinctipes* individuals respond to thermal stimulus isolated to their walking legs. This provides evidence that suggests that crabs have thermosensory systems in their walking legs that could be triggering heat avoidance behavior. Understanding how organisms generate and integrate thermosensory information to accurately perceive and respond to their environment is important because thermotactic guided behavior contributes to thermoregulation to effectively avoid lethal temperatures (Harshaw et al., 2017).

Gravid and non-gravid females did not differ in neural thermal sensitivity (Figure 9). This could be due to the seasonality of sampling and the fact that the NGF may have been reproductive but had not extruded their broods yet. Although the gravid state has a significant effect on VT_max_ (Figure 7), a larger sample size and collection throughout the year may be needed to link the effects of gravidity to the relationship between neural and whole organism sensitivity to temperature. Nonetheless, there was a significant positive correlation between peak nerve firing temperature and VT_max_ among all crabs (Figure 10). Although the neural thresholds for peak firing temperature are higher than escape temperature, they are in line with the temperatures that elicited a response in isolated thermal stimulus experiments (Figure 8). The effect of temperature on neural systems corresponds tightly with thermal thresholds that trigger behavior, suggesting that the thermosensitive property of neurons in the walking leg may result in motor output responsible for behaviorally determined temperature selection. This finding provides evidence for thermosensitive neurons acting as a mechanistic trigger for whole organism behavior. There is compelling evidence linking thermosensory neurophysiology and thermoregulatory behavior in porcelain crabs.

## Conclusion

The extensive fine-scale thermal variability present along a boulder shore intertidal gradient is likely to influence the distribution and demographics of taxa through life-cycle dependent physiological and behavioral processes. To examine if demographic patterns could be partially explained by interactions between temperature-dependent behavior and reproductive state, this study integrated demographic, molecular, behavioral, and physiological approaches. Most notably, the results suggest that behavioral responses to temperature depend on female reproductive state. Specifically, gravid females became more heat sensitive, which could influence species demographics and ranges under warming conditions. As warming occurs, the most fecund individuals may move to cooler areas first, leaving warm edge populations with fewer reproductive individuals. Furthermore, changes in behavioral responses may be mediated by variation in afferent neuron temperature sensitivity, suggesting a locus of interest for further study into the mechanisms underlying species responses to warming. Overall, this study reinforces that an integrative research program that spans multiple approaches and levels of organization can yield important insights into ecological processes under anthropogenic global change.

## Data Availability Statement

The data presented in the study are deposited in the Dryad repository, accession number https://doi.org/10.6078/D1G994.

## Author Contributions

EL, MA, and AG: conceptualization, formal analysis, investigation, methodology, and writing—original draft. EL, MA, and AG: data curation. AG, BT, and JS: project administration and supervision. JS and BT: resources. EL, MA, AG, and JS: writing—review and editing. All authors gave final approval for publication and agreed to be held accountable for the work performed therein.

## Conflict of Interest

The authors declare that the research was conducted in the absence of any commercial or financial relationships that could be construed as a potential conflict of interest.

## Publisher’s Note

All claims expressed in this article are solely those of the authors and do not necessarily represent those of their affiliated organizations, or those of the publisher, the editors and the reviewers. Any product that may be evaluated in this article, or claim that may be made by its manufacturer, is not guaranteed or endorsed by the publisher.
