# Supplementary material for: Interactions Between Temperature Variability and Reproductive Physiology Across Traits in an Intertidal Crab
Source: Front Physiol. 2022 Mar 8;13:796125. doi: 10.3389/fphys.2022.796125 (PMC8957995; doi:10.3389/fphys.2022.796125)
Supplement: Supplementary file 4 [file Data_Sheet_2.docx]

Supplementary Material

# Supplementary Figures


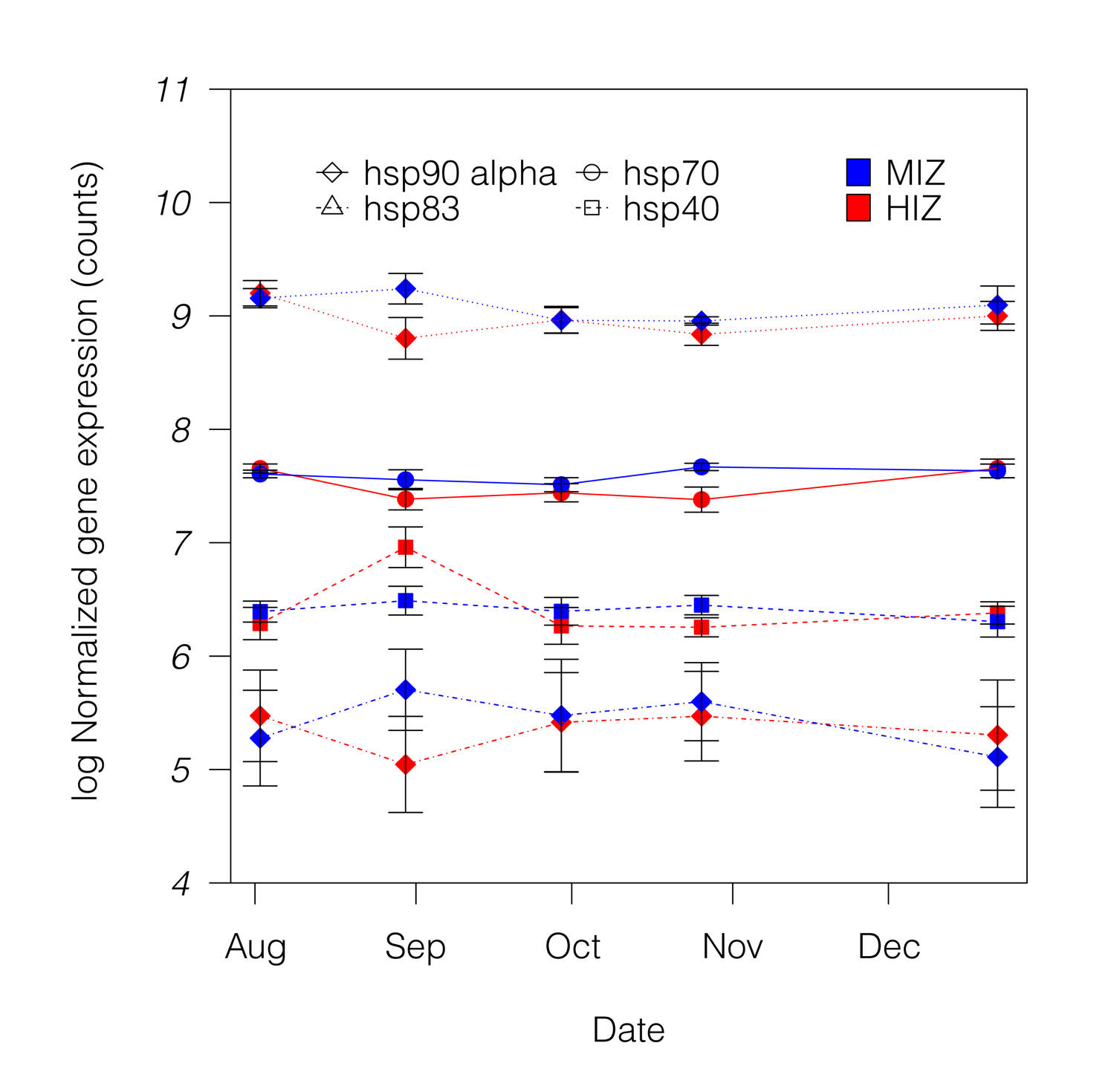


**Supplementary Figure S1.** Log normalized gene expression of heat shock proteins related to date and intertidal zone height.


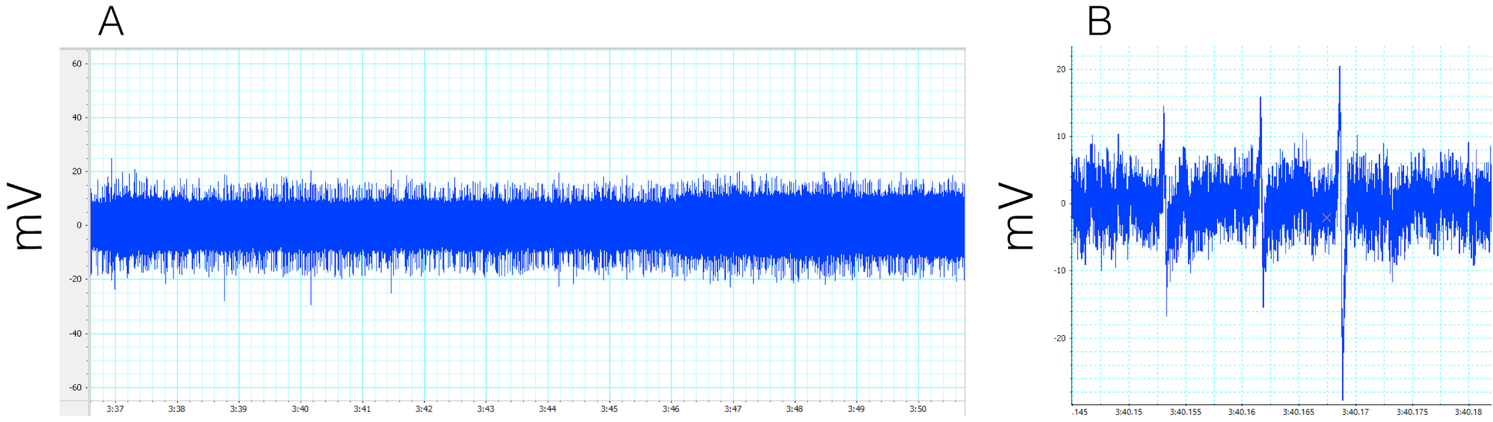


**Supplementary Figure S2.** Raw data recording of field potentials from an individual crab in millivolts (mV) over time (minutes: seconds. milliseconds) a sampling rate of 40k/s. during a thermal ramp at 17ºC using LabChart software. (B) is a magnified view of action potentials from (A).

# Supplementary Tables

**Supplementary Table S1**. (A) Probe sequences, (B) raw and (C) normalized Nanostring data from female and male crabs according to intertidal zone height and sampling date.

**Supplementary Table S2**. Summary statistics for gene expression principal components analysis, including PC loadings for each gene (from the *prcomp* function in R), standard deviation of each PC axis, variation explained by each PC axis, and cumulative variation explained by the PC axes.

# Supplementary Videos

**Supplementary Video 1.** An example of a run during the behavioral heat avoidance experiment as a time lapse video. A crab is escaping the temperature chamber and has a (VT_max_) of 23.6ºC.

**Supplementary Video 2.** Video of an early iteration of the behavioral responses to isolated thermal stimulation experiment. Ambient (13ºC) and hot (40ºC) water was administered to the leg of a crab with a transfer pipette. The crab did not move away in response to the ambient thermal stimulus but moved away in response to the hot thermal stimulus. The actual experiment was conducted as stated in the methods with a syringe and one drop of water at discreet temperatures between 17-39ºC.

**
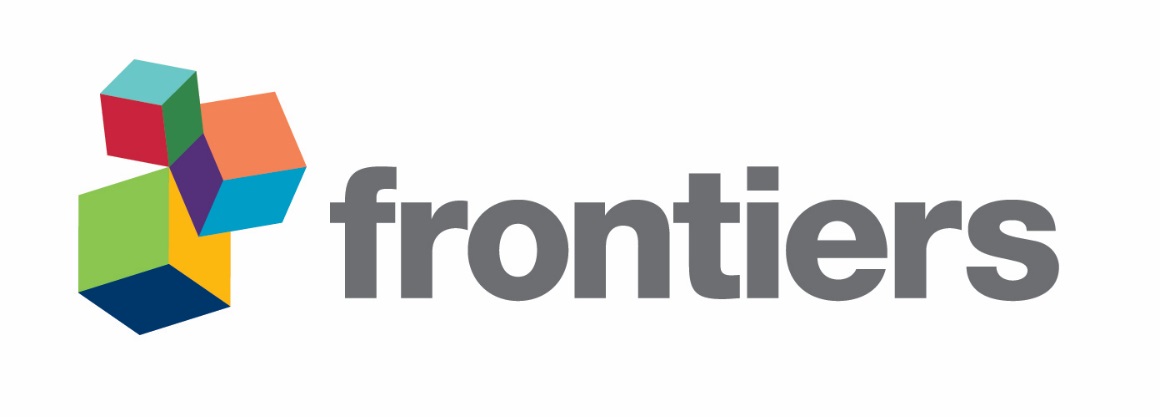
**
